# RobotP: A Benchmark Dataset for 6D Object Pose Estimation

**DOI:** 10.3390/s21041299

**Published:** 2021-02-11

**Authors:** Honglin Yuan, Tim Hoogenkamp, Remco C. Veltkamp

**Affiliations:** Department of Information and Computing Sciences, Utrecht University, 3584 CC Utrecht, The Netherlands; thoogenkamp@gmail.com (T.H.); R.C.Veltkamp@uu.nl (R.C.V.)

**Keywords:** benchmark dataset, 6D pose estimation, sensors, 3D reconstruction

## Abstract

Deep learning has achieved great success on robotic vision tasks. However, when compared with other vision-based tasks, it is difficult to collect a representative and sufficiently large training set for six-dimensional (6D) object pose estimation, due to the inherent difficulty of data collection. In this paper, we propose the RobotP dataset consisting of commonly used objects for benchmarking in 6D object pose estimation. To create the dataset, we apply a 3D reconstruction pipeline to produce high-quality depth images, ground truth poses, and 3D models for well-selected objects. Subsequently, based on the generated data, we produce object segmentation masks and two-dimensional (2D) bounding boxes automatically. To further enrich the data, we synthesize a large number of photo-realistic color-and-depth image pairs with ground truth 6D poses. Our dataset is freely distributed to research groups by the Shape Retrieval Challenge benchmark on 6D pose estimation. Based on our benchmark, different learning-based approaches are trained and tested by the unified dataset. The evaluation results indicate that there is considerable room for improvement in 6D object pose estimation, particularly for objects with dark colors, and photo-realistic images are helpful in increasing the performance of pose estimation algorithms.

## 1. Introduction

Six-dimensioal (6D) pose estimation is crucial for many vision-based applications, such as visual navigation, robot manipulation, and virtual reality [1,2,3,4]. The awareness of the three-dimensional (3D) rotation and 3D translation matrices of objects in a scene is referred to as 6D, where the D stands for degrees of freedom pose. However, estimating object poses is challenging, for objects in the real world have various shapes, sizes, and textures. On the other hand, even though increasing algorithms aiming to estimate the 6D object pose have been published, different pose estimation methods have different strengths and weaknesses, and it is unclear how well they perform, due to the lack of benchmarks with high-quality datasets.

While it is possible to obtain the 6D pose by handcrafted feature-based methods [5,6], these approaches fail to predict poses for texture-less or reflective objects, as they are easily affected by sensor noise, changing lighting conditions, and occlusion. With the advent of cheap RGB-D sensors, the precision of 6D object pose estimation is improved significantly [3]. Nonetheless, it remains a challenge, as the heavy dependence on handcrafted features and fixed matching process has limited the empirical performance of these methods.

Recent studies [7,8,9,10,11] show that learning-based methods are able to produce results that are comparable to or even better than classical state-of-the-art methods. Instead of relying on handcrafted features, they learn more robust features and semantic cues by applying deep learning models. More specifically, learning-based approaches often use Convolutional Neural Network (CNN) based architectures to extract visual and geometric features from RGB-D images and fuse these features together. Subsequently, the fused features are used to directly predict the 6D object pose. Their performance [1,12,13] has been reported on the LineMOD [14] and YCB-Video [9] datasets. However, current datasets that are designed for pose estimation have several limitations: (1) the objects are often located in the center of the image plane and the captured images have limited viewpoints and low resolution; and, (2) generating datasets has a high cost (time and money) that is associated with ground truth annotation.

**Figure 1:**
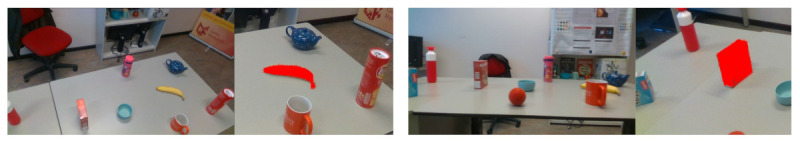
Scene examples and visualization of estimated poses by the approach that was proposed from our benchmark.

To address these challenges, we organize the Shape Retrieval Challenge benchmark on 6D pose estimation and create a new benchmark dataset that is representative enough for the pose estimation problem and also contains a considerable amount of variability for training and testing learning-based 6D object pose estimation algorithms, as shown in Figure 1.

Generating 6D object pose estimation dataset presents specific challenges. The first challenge is selecting and modeling objects that are suitable for benchmarking 6D object pose estimation performance. Research groups often select objects based on the aims they plan to achieve [15]. Consequently, the selected objects perhaps do not cover various pose estimation challenges, and not be available to other researchers (e.g., they are only available in certain regions). To address these problems, we take several practical issues into consideration when selecting objects, such as the size, cost, and characteristic of the object. In order to generate high-quality 3D models, we first use a well-chosen 3D camera to collect RGB-D images and then propose generating the 3D model for each object by an image-based 3D reconstruction approach.

The second challenge is to provide high-quality RGB-D images, ground truth poses, segmentation masks, and 2D bounding boxes for each object. 3D cameras allow easy 3D acquisition of objects, but have key limitations. For example, the captured depth images often have missing data and they do not align well with their corresponding color images. Even though depth recovery algorithms [16,17] provide aligned depth images, they fail in occlusion regions when the camera is near the object. In contrast, multi-view stereo (MVS) can achieve better results for these regions. Taking advantage of these two kinds of depth images, we propose a novel depth generation approach to create high-quality depth images by aligning and fusing them.

The Structure from Motion (SfM) [18], which is based on feature matching to estimate 6D poses, is often used to generate ground truth poses. A fundamental limitation of SfM is that it is unable to provide accurate poses when the change between two cameras becomes larger. In order to address this problem, a pose refinement approach combining local and global pose optimization is introduced. Besides, object mask and 2D bounding box annotation is a time-consuming and expensive process, as humans often generate the annotation [19]. Instead of relying on humans, we propose a novel method to generate accurate segmentation masks and 2D bounding boxes automatically and cost-effectively.

The third challenge is generating large numbers of scene images that were captured in a variety of viewpoints. Collecting real-world data is a tedious and labor intensive process. When compared with datasets that were produced by real-world data, synthesizing such a dataset requires less hardware, time, and human labor, while it is more likely to result in better quality. Taking advantage of image based rendering that can provide the free-viewpoint and realistic imagery of real scenes, we generate a large number of reasonable and photo-realistic images with ground truth 6D poses. Even though we use synthesized images, they are still useful, as the synthesized images are photo-realistic, which are able to bridge the reality gap that allows models trained with synthetic data to the real world without domain adaption.

Our main contributions are summarized, as follows:

- A representative dataset providing high-quality RGB-D images, ground truth poses, object segmentation masks, 2D bounding boxes, and 3D models for different objects, which covers a wide range of pose estimation challenges.
- A novel pose refinement approach that uses a local-to-global optimization strategy to achieve the improved accuracy of each pose and global pose alignment.
- A novel depth generation algorithm producing high-quality depth images, which is able to accurately align the depth image to its corresponding color image and fill missing depth information.
- Careful merging of multi-modal sensor data for object reconstruction, followed by an algorithm to produce the segmentation mask and 2D bounding box for each object automatically.
- A training dataset is generated by a free-viewpoint image based rendering approach in a simulated environment. It provides a large amount of high-resolution and photo-realistic color-and-depth image pairs that have plausible physical locations, lighting conditions, and scales.
- The Shape Retrieval Challenge benchmark on 6D object pose estimation. The benchmark allows for evaluating and comparing pose estimation algorithms under the same standard. The evaluation results indicate that there is considerable room for improvement in 6D object pose estimation, particularly for objects that have dark colors or reflective characteristics, and knowledge that is learned from photo-realistic images can be successfully transferred to real-world data without domain constraints.

## 2. Related Work

Prior works collect plenty of datasets for vision-based applications, such as object detection [20] and image classification [21,22,23], as well as for benchmarking in 3D shape retrieval [24,25]. However, few datasets are available for 6D object pose estimation which plays an important role in robotic grasping and manipulation. The LineMOD dataset [14] and KIT Object Models Database [26] are the earliest 3D datasets for 6D object pose evaluation. The LineMOD dataset contains 13 texture-less objects of varying shapes and sizes, and the objects are captured under different lighting conditions. However, the captured images in it have limited viewpoints. The KIT Object Models Database consists of 2D images and 3D mesh models of over 100 objects which are obtained semi-automatically. Even though the number of objects in this dataset is large, the objects are not easily accessible to other researchers, due to regional product differences. Later, the Linemod-Occluded dataset [27] is proposed to address occlusion problem, which is a subset of the LINEMOD dataset. The RGB-D images provided by this dataset contain multiple annotated objects that are heavily occluded. The IC-MI dataset [28] is introduced to further investigate the performance of pose estimation in heavily cluttered and occluded scenes. It has two texture-less and four textured household objects and its testing images have multiple objects containing heavy 2D and 3D clutters, and foreground occlusion. The IC-BIN dataset [29] provides 3D models and RGB-D images of two objects from IC-MI. It is designed for a bin-picking scenario, which also contains objects in the heavy occlusion condition. A characteristic of this dataset is that it includes multiple instances of the same object. However, clutters and occlusion in this dataset are moderate, which makes it not particularly challenging.

Rutgers APC dataset [30] is specifically designed for solving warehouse pick-and-place tasks. It consists of 24 different objects that are placed on a cluttered warehouse shelf from the Amazon Picking Challenge 2015 [31]. The T-LESS [32] and ITODD [15] datasets focus on industry-relevant objects. The objects in the T-LESS dataset have no significant texture and discriminative color or reflectance properties. They exhibit symmetries and mutual similarities in shape and/or size. Similarly, ITODD dataset [15] contains 28 objects with 3D models, which are captured in realistic industrial setups. When compared to the T-LESS dataset [32] , it features objects with a special focus on planarity, size, and complexity. The main disadvantages of these datasets are that the objects are often located in the center of the image plane, which limits the richness of viewpoints, and data annotation for these datasets is tedious and labor-intensive. In contrast, our dataset contains high-quality RGB-D images that are captured from a variety of viewpoints and labeled automatically.

With the development of data-driven methods designed for robotics applications [33], the importance of synthetic data has been highlighted. Recent works [8,9,34,35,36] combine real and synthetic data to generate 3D object datasets, which render 3D object models on real backgrounds in order to produce synthesized images. YCB-Video [9] dataset is the mostly used 3D object datasets for 6D object pose estimation. It contains 21 objects with different shapes and textures. Apart from the captured images, synthesized images are also provided, which are produced by projecting 3D models to arbitrary backgrounds. While the backgrounds are realistic, the synthesized images are not reasonable (e.g., objects are often flying in midair [35]). Unlike the YCB-Video dataset, objects in the TYO-L dataset [37] are captured on a table-top setup, with four different table cloths and five different lighting conditions. The objects in this dataset are rendered with fixed lighting conditions and a black background to generate the synthetic training images. Unlike these methods, we are able to mimic the physical behavior of the camera and provide reasonable and photo-realistic images by image based rendering in a simulation environment.

Our dataset could be considered to be between LineMOD and YCB-Video datasets. It contains high-resolution RGB-D images, high-quality annotations, and a large number of photo-realistic synthesized images. In contrast to the recent work of Hodan et al. [37], which combines eight different datasets for benchmarking 6D object pose estimation, we particularly focus on the domain adaption for photo-realistic synthetic data. Table 1 summarizes the object datasets that have been proposed for the 6D object pose estimation task.

## 3. The RobotP Dataset

Our goal is to build a benchmarking dataset that allows for evaluating and comparing the performance of different 6D pose object pose estimation methods under the same standard. We aim to cover as many pose estimation challenges as possible, including occlusion, poor lighting conditions, and varying viewpoints, shapes, and textures, with a special focus on the effect of training images.

The dataset generation works, as follows: we first select eight representative and daily used objects with the consideration of many practical issues (Section 3.1). Subsequently, we collect real-world data for these objects by a well-chosen 3D camera under different scenarios (Section 3.2). Next, from the collected data, we estimate ground truth 6D poses for these objects (Section 3.3) and generate high-quality depth images (Section 3.4). After that, we reconstruct textured 3D models and, based on the 3D models, we generate object masks and two-dimensional (2D) bounding boxes automatically (Section 3.5). Furthermore, to augment the collected data, we synthesize a large number of photo-realistic images with ground truth 6D poses (Section 3.6).

### 3.1. Object Selection

The first step of generating the RobotP dataset is to choose objects that are frequently used in daily life. A large number of objects in a 3D dataset is necessary for addressing pose estimation challenges. However, the quantity of the objects is not sufficient for building a representative 3D dataset. To make our dataset more general, when selecting objects, several issues have been considered:

1. In order to cover as many aspects of pose estimation challenges as possible, the selected objects should be representative enough for the pose estimation problem. For example, objects with few textures are added to the dataset, as it is a challenge for pose estimation approaches to estimate 6D poses for texture-less objects is representative enough for the pose estimation problem.
2. We aim to provide a 3D dataset allowing for easily carrying, shipping, and storing, which is helpful to carry out robotic manipulation experiments in the real world. Thus, the portability of the object is taken into consideration.
3. To make the dataset easily reproducible, we choose the popular consumer products, which are low price and easy to buy as our target objects.

**Figure 2:**
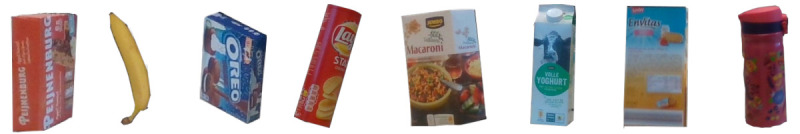
Daily used objects in our dataset.

With consideration of these practical issues, we finally select eight representative objects to create our dataset, as shown in Figure 2.

### 3.2. Collecting Scene Data

The second step is to collect a set of color-and-depth image pairs that are able to represent the selected objects. Instead of using capture rigs, we use a hand-held 3D camera to collect data, for it has the close resemblance to regular cameras, and it has the ability to introduce more variation to camera poses. To choose the 3D camera, three main issues should be considered:

- The resolution of the captured image should be as high as possible. This is because, with high resolution images, we are able to obtain richer information about the captured object.
- The range of the 3D camera should be long enough, which allows for us to capture images with a variety of view positions; The frame rate of the 3D camera should be high, which is able to promise better tracking of capture processes.
- The 3D camera should be portable and low-cost, allowing large groups of inexperienced users to collect data.

**Figure 3:**
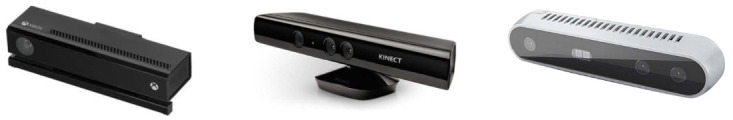
Different three-dimensional (3D) cameras. Left: time-of-flight camera. Middle: structured-light camera. Right: depth-from-stereo camera.

There are three main types of 3D cameras: time-of-flight, structured-light, and depth-from-stereo 3D cameras, as shown in Figure 3. A detailed summary of the available consumer 3D cameras can be found in [38]. After analyzing the practical issues that are mentioned above, we choose Intel RealSense D415 camera that is based on depth from stereo to generate depth images as our target camera (the camera on the right in Figure 3). It provides more accurate depth perception and longer range, and it uses an infrared projector to improve the depth perception ability for texture-less scenes. Table 2 describes its basic features.

We acquire RGB-D videos by the Intel RealSense D415 camera that is connected to a laptop that allows recording RGB-D videos for several hours. Depth and color frames are captured with a resolution of 1280×720. For camera calibration, we use its default parameters, as Intel has its own calibration system that has advances over the free calibration software. It has the ability to calibrate both extrinsic and intrinsic parameters, and calibrate multiple cameras simultaneously. Apart from calibration, we use the Intel “High Density” setting for depth calculation. This is because it provides us with better quality depth images containing few holes and allows us to capture as much data as possible in all of the depth ranges.

### 3.3. Ground Truth Pose Estimation

**Figure 4:**
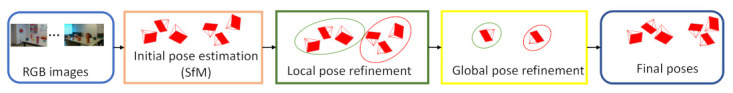
The pipeline of the pose estimation process. The input are RGB images and the initial poses of these images are estimated by Structure from Motion (SfM). After that, the initial poses are refined locally and globally.

Unlike previous approaches requiring estimating a markerboard pose to obtain ground truth poses, we directly estimate camera poses by Structure from Motion (SfM) [18]. However, the estimated camera pose is more likely to have errors because of the inherent limitation of SfM, when the change between two input images becomes larger or the images do not have enough features. In order to improve the accuracy of estimated poses, we perform pose refinement that combines local and global pose refinement steps. Instead of refining all camera poses simultaneously, we first divide poses into groups and then refine poses in each group. After that, we choose a key pose from each group and then refine these key poses globally. Our aim is to respect the local details and also be compatible with global consistency. Figure 4 describes the pipeline of pose estimation.

**Figure 5:**
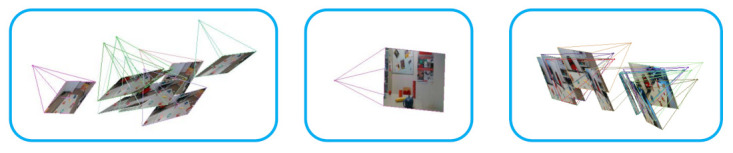
The local pose groups. They are clustered based on angle and distance similarities.

In the pose refinement process, poses are first grouped based on their similarities among each other. The similarity is measured by comparing the angle and distance between two poses. We randomly define one pose as our key pose and then calculate the angle and distance between the key and other poses. We first rank the poses based on the distances that they have with the key pose. Next, we check if their corresponding angles are bigger than the field of view of the camera. If so, we then delete the pose from the rank. After that, we choose up to ten top poses as a local group. Subsequently, the other groups are obtained by the same pipeline from the remaining poses. Figure 5 shows the clustered groups that are used for pose refinement.

We use bundle adjustment to refine camera poses in the local group with the consideration of its neighbors. Bundle adjustment [39] method is often used in 3D reconstruction as the joint non-linear refinement of camera parameters and feature points. We choose the key pose’s corresponding image as the key image, and then we detect feature points between the key image and other poses’ corresponding images. For each image, scale-invariant feature transform (SIFT) features are detected and matched. The reason to use SIFT feature is that it is robust for the major variation, such as image translation, scaling, and rotation. Next, we project these feature points into the 3D world space. Lastly, we apply local bundle adjustment to refine camera poses with these 3D points. To account for potential outliers, the Huber function is used as the robust loss function in local bundle adjustment, and we use Ceres Solver library to solve the optimization function. The cost function for grouped images is defined as:

(1) $$\frac{1}{2}\sum_{i=1}^{m}\sum_{j=1}^{n}\left|\right|e_{i,j}{\left|\right|}^{2}=\frac{1}{2}\sum_{i=1}^{m}\sum_{j=1}^{n}||f\left(P_{j},x_{i}\right)-X_{j}{\left|\right|}^{2},$$

where $e_{i,j}$ is the reprojection error and $P_{j}$ is the projection matrix. Assume that *n* 3D points are seen in *m* views, function *f* projects point $x_{i}$ in the image plane to 3D world space, and $X_{j}$ is the reference point in the world space.

After the local pose refinement, we build a feature group by computing the features from key images for global pose refinement. However, the feature group may contain multiple instances of the same real-world point that are found in separate pairwise image matches. To address this issue, we only add features which have been used in local pose optimization process to the feature group. We then compute the 3D positions of these features based on the optimized poses. Once the feature points and their corresponding 3D points are obtained, we use the same loss function as local pose optimization to refine these global poses.

### 3.4. Depth Generation

**Figure 6:**
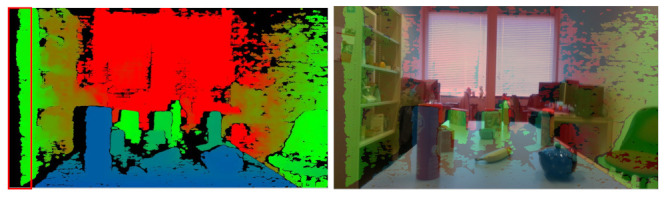
(**a**) The captured depth image: the red rectangle shows left invalid depth band. (**b**) Misalignment of color-and-depth image pairs: the images are generated when the distance between the object and camera is near, showing large misalignment.

Color images have been successfully used by deep learning for many robotic vision tasks, such as object recognition and scene understanding. However, grasping objects with the exact physical dimensions is a very hard problem that requires not only RGB data, but also extra information. Depth images can provide such a brand-new channel of information and are essential elements in the datasets designed for 6D pose estimation. However, captured depth images often suffer from missing information and misalignment between color-and-depth image pairs due to the inherent limitation of depth cameras. The Intel RealSense D415 camera also has the same limitation. Even though the alignment and hole filling methods from Intel are applied, the quality of the captured depth image is still low, especially when the camera is near the object (see Figure 6). Therefore, new algorithms are required to improve the quality of captured depth images.

#### Depth and Color Image Alignment

Because the Intel RealSense D415 camera is based on depth from stereo to calculate depth values, it uses the left sensor as the reference for stereo matching. This leads to a non-overlap region in the field of view of left and right sensors, where no depth information is available at the left edge of the image (see Figure 6a).

Based on the stereo vision, the depth field of view (DFOV) at any distance (*Z*) can be defined by [40]:

(2) $$DFOV=\frac{HFOV}{2}+\tan^{-1}\left(\tan\frac{HFOV}{2}-\frac{B}{Z}\right),$$

where HFOV is the horizontal field of view of left sensor on the depth module and *B* is the baseline between the left and right sensors.

We can see that, when the distance between the scene and the depth sensor decreases, the invalid depth band increases, which results in the increase of the invalid depth in the overall depth image. Besides, if the distance between the object and the depth sensor decreases, the misalignment between the color and depth image also increases, as shown in Figure 6b.

In previous works, a new depth image is created, which has the same size as the color image but the content being depth data calculated in the color sensor coordinate system, in orde to align the depth image to its corresponding color image. In other words, to create such a depth image, the projected depth data is determined by transforming the original depth data to the color sensor coordinate system based on the transformation matrix between the color and depth sensors. However, it is difficult to obtain the correct transformation matrix, as the depth and color images are defined in different spaces and have different characteristics.

**Figure 7:**
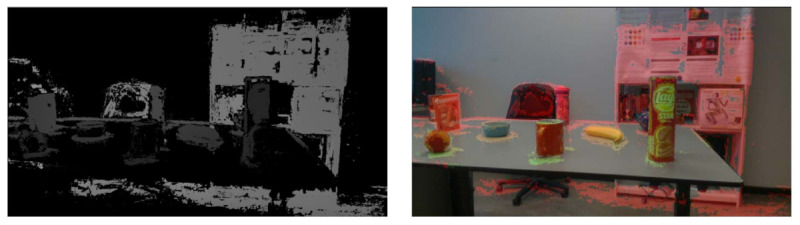
The depth images are estimated by COLMAP, showing better alignment.

To solve this problem, we first create an estimated depth image for each color image by MVS from COLMAP [41]. The estimated depth image has better alignment with the color image (see Figure 7), as it is estimated with the consideration of photometric priors and global geometric consistency. Subsequently, we align the captured depth images to estimated depth images to achieve better alignment between color-and-depth image pairs. For the captured and estimated depth images have the same characteristics, it is easier for us to align the captured depth image to the estimated depth image.

In order to find correspondences between captured and estimated depth images, we compare depth values and normals between them. We should make sure the estimated and captured depth images have the same scene scale in order to compare depth values. However, a fundamental limitation of the estimated depth image is that we do not know the scale of the scene. We use linear regression in a random sample consensus (RANSAC) loop to find the metric scaling factor. After obtaining the scaling factor, we use it to scale the estimated depth image to the captured depth image.

We convert the depth image to a point cloud by camera intrinsic matrix to estimate normals. Subsequently, we compute the surface normal at each point in the point cloud. Determining the normal to a point on the surface can be considered as estimating the normal of a plane tangent to the surface. Thus, this problem becomes a least-square plane fitting estimation problem [42]. Let *x* be a point and $\vec{n}$ be a normal vector. The plane is represented as $\pi (x,\vec{n})$. The distance from a point $q_{i}$ in a point set *Q* to the plane π is defined by $d_{i}=\left(q_{i}-x\right)\cdot \vec{n}$. Because the values of *x* and $\vec{n}$ fit the least-square sense, $d_{i}=0$. Subsequently, we define *x* as the centroid of *Q*:

(3) $$x=\frac{1}{k}\sum_{i=1}^{k}q_{i},$$

where *k* is the number of points in *Q*. Therefore, the solution for estimating the normal $\vec{n}$ is reduced to analyze the eigenvectors and eigenvalues of a covariance matrix *C* created from *Q*. More specifically, the covariance matrix *C* is expressed as:

(4) $$C=\frac{1}{k}\sum_{i=1}^{k}\alpha_{i}\left(q_{i}-x\right){\left(q_{i}-x\right)}^{T},\;C\cdot \vec{v_{j}}=\beta_{j}\cdot \vec{v_{j}},j\in \left\{0,1,2\right\},$$

where $\alpha_{i}$ is a possible weight for point $q_{i}$, $\vec{v_{j}}$ is the *j*-th eigenvector of the covariance matrix, and $\beta_{j}$ is the *j*-th eigenvalue. The normal $\vec{n}$ can be computed based on (4).

Our aim is to produce better aligned color-and-depth pairs for objects not the overall scene, as the generated dataset is used for object pose estimation. We first extract a patch $Pa_{c}$ containing a target object in the captured depth image $D_{c}$. Afterwards, we define an offset map whose size is the same as $Pa_{c}$ but the content being index differences between $Pa_{c}$ and its corresponding patch in the estimated depth image $D_{e}$. In ideal conditions, the values in the offset map should be zeros.

The matching process, which is based on PatchMatch [43], is implemented by first initializing the offset map with random values. Subsequently, we extract a patch $Qb_{e}$ that is based on the offset map as the corresponding patch for $Pa_{c}$. The pixel p(x,y) in $Pa_{c}$ is transformed to pixel $q(x^{\prime },y^{\prime })$ in $Qb_{e}$ by:

(5) $$q\left(x^{\prime },y^{\prime }\right)=p\left(x+x_{off},y+y_{off}\right),$$

where $\left(x_{off},y_{off}\right)$ is the index offsets for each pixel in $Pa_{c}$.

After that, we perform an iterative process which allows good index offsets propagating to its neighbors to update the offset map. The iteration starts with the top left pixel and then an odd iteration starting with the opposite direction. We first calculate the depth differences $dd_{i}$ between pixel $a_{i}\in Pa_{c}$ and pixel $b_{i}\in Qb_{e}$, and the angles between normals $\vec{n_{a_{i}}}$ and $\vec{n_{b_{i}}}$. If the angle is smaller than a predefined threshold, then $dd_{i}$ is saved. Subsequently, if $dd_{i}$ is smaller than its neighbors, we replace the offsets of $a_{i}$’s neighbors with $a_{i}$’s offset. After every iteration, we calculate the sum $c_{i}$ of $dd_{i}$. We stop propagation when the change of $c_{i}$ is negligible. Finally, we map the captured depth image to the estimated depth image based on the offset map. Algorithm 1 summarizes the depth alignment process.

**Algorithm 1** Overview of depth alignment procedure.**Input** Captured depth image $D_{c}$, estimated depth image $D_{e}$;**Output:** aligned depth map $D_{c1}$ for $D_{c}$;  1: Run RANSAC to find the metric scaling factor.  2: Extract patch $Pa_{c}$ in $D_{c}$.  3: Calculate scaled depth values and normals of $Pa_{c}$.  4: Initialize offset map *O*.  5: Find a patch $Qb_{e}$ in $D_{e}$ based on *O*.  6: **for**
$a_{i}$ ∈ $Pa_{c}$ and $b_{i}$ ∈ $Qb_{e}$
**do**  7:    Calculate depth difference $dd_{i}$ and normal angle $ang_{i}$ between $a_{i}$ and $b_{i}$.  8:    Run PatchMatch propagation to update offset map *O*.  9: Mapping $D_{c}$ to $D_{c1}$ based on *O*.

#### 3.4.2. Depth Fusion

Even though the captured depth image is aligned to its corresponding color image, the invalid depth band still exists. Apart from that, it has missing information and noise, especially when reflective or transparent objects are captured. On the other hand, the estimated depth image generated by MVS not only has better alignment with its corresponding color image, but it also provides useful depth information in regions where the depth camera has poor performance. However, the estimated depth image is not able to provide reliable depth information for texture-less or occluded objects, due to the inherent limitations of MVS. Thus, the quality of the estimated depth image is not sufficient for our dataset either.

Because the characteristics of the captured and estimated depth images are complementary, we fuse the captured and estimated depth images together to create a fused depth image. The fused depth image takes advantage of both real captured and estimated depth images, resulting in the improved quality. We generate the fused depth image $D_{f}$ by the maximum likelihood estimation:

(6) $$D_{f}=\underset{d}{argmax}\left(\left(R_{e}P_{e}\right)\left(R_{c}P_{c}\right)\right),$$

where *d* is the depth value, $R_{e}$ is a reliability map and $P_{e}$ is a probability map produced from the estimated depth image, and $R_{c}$ is a reliability map and $P_{c}$ is a probability map that is produced from the captured depth image.

The reliability map $R_{c}$ for the captured depth image is computed according to the variation between the depth value and camera’s range. The reliability $r_{c}$ of each depth value *d* is calculated by

(7) $$r_{c}=\left\{\begin{array}{cc} \frac{MaxD^{2}-d^{2}}{MaxD^{2}-MinD^{2}}, & MinD<d<MaxD\\ 0, & otherwise \end{array},\right.$$

where MaxD and MinD are the minimum and maximum distances that the depth camera is able to measure. From (7), we can see that when the distance between the camera and the scene increases, the precision decreases. After calculating the reliability for each pixel, we obtain the reliability map $R_{c}$.

We take the depth image generation into consideration in order to obtain the reliability map $R_{e}$ for the estimated depth image. The estimated depth image is generated based on COLMAP, which runs in two stages: photometric and geometric. The photometric stage only optimizes photometric consistency during depth estimation. In the geometric stage, a joint optimization, including geometric and photometric consistency, is performed, which can make sure the estimated depth maps agree with each other in space. We obtain the reliability $r_{e}$ of each depth value by comparing the depth values $d_{p}$ and $d_{g}$ computed from photometric and geometric stages, respectively:

(8) $$r_{e}=\left\{\begin{array}{cc} \frac{\delta -|d_{g}-d_{p}|}{d_{g}}, & d_{g}<\left|d_{g}-d_{p}\right|\\ 0, & otherwise \end{array},\right.$$

where δ is the maximum accepted depth difference that is set to be 50 in our experiments. When the depth value calculated based on geometric consistency has a large difference when compared with the depth value calculated based on photometric consistency, we consider this value is unreliable.

One of the main limitations of the reliability map is that it does not take the idea that spatial neighboring pixels are able to be modeled by similar planes into account. We introduce the probability map in order to solve this problem. To calculate the probability of a depth value in the captured or estimated depth image, we define a (5×5) support region *S* centered at the pixel *i* whose depth value is $d_{i}$. For each pixel j∈S, if *j* is far from *i*, then it is reasonable to associate a low contribution to *j* when calculating the probability for $d_{i}$. Following this intuition, the probability $p_{d_{i}}$ is estimated by

(9) $$p_{d_{i}}=\sum_{j\in S}e^{-\frac{\Delta_{i,j}}{\gamma_{1}}}\cdot e^{-\frac{\Delta_{i,j}^{\pi }}{\gamma_{2}}},$$

where $\Delta_{i,j}$ is the euclidean distance between *i* and *j*, $\Delta_{i,j}^{\pi }$ calculated by (4) accounts for the distance from *j* to the plane π, and $\gamma_{1}$ and $\gamma_{2}$ control the behavior of the distribution. We empirically set $\gamma_{1}$ and $\gamma_{2}$ to be 4 and 1, respectively. We produce the probability maps $P_{e}$ and $P_{c}$ after calculating the probability for every depth value in the estimated and captured depth images, respectively.

Finally, with the reliability and probability maps, we generate high-quality fused depth images that are based on (6).

### 3.5. 3D Modeling

We use COLMAP, which is based on a collection of RGB images for 3D modeling, to generate the 3D point cloud for each object. However, it fails on some objects with fewer features, such as transparent or texture-less objects. To obtain more accurate 3D point clouds, we use depth images that were generated in Section 3.4 to refine the initial model, as they provide reliable depth information in regions with missing features.

To refine the initial point cloud, we project a pixel in the color image to its neighboring images to check whether it is visible in them. If it is visible in more than five images, we project this pixel to the world coordinate system to get the 3D point which is added to the initial 3D point cloud. Then we check if there are many similar 3D points around it. If so, we will not add this point into the 3D point cloud, for we only save key points in our 3D point cloud. After all of the pixels in the image are projected, we remove outliers that are often caused by measurement errors, boundaries of occlusion, or surface reflectance by the StatisticalOutlierRemoval filter from Point Cloud Library (PCL) [44]. This filter performs a statistical analysis on the neighboring points of each point. Supposing that the filtered point cloud is Gaussian distribution, all points whose mean distances are outside an interval defined by the global distance mean and standard deviation can be considered as outliers and trimmed from the point cloud. We repeat the previous steps until all of the images are projected.

**Mask and bounding box generation.** Apart from 3D models, we also provide masks and corresponding 2D bounding boxes for the objects in our dataset. Our goal is to generate accurate segmentation masks and 2D bounding boxes automatically and cost-effectively. In order to achieve our goal, we take three practical issues into consideration:

- Quality. Each mask and its corresponding bounding box need to be tight. For example, the bounding box should be the minimum bounding box that fully encloses all visible parts of the object. In order to estimate the 6D object pose, the first step is to detect the target object. The quality of masks and bounding boxes influences the performance of object detection algorithms, which affects the accuracy of the estimated pose.
- Coverage. Each object instance needs to have a segmentation mask and a 2D bounding box that should only contain the object other than the background. For learning-based object detection and recognition approaches, they need to know exactly which part is the target object in a whole image.
- Cost. The designed algorithm should not only provide high-quality masks and 2D bounding boxes, but also have the minimum cost, as data annotation is a labor intensive and time-consuming process.

We take advantage of the generated 3D point cloud and 6D poses to address these issues. To produce the segmentation mask, we project the point cloud into each image plane while using the estimated 6D pose and camera intrinsic matrix. In this way, our goal can be achieved easily. After that, we obtain the 2D bounding box by detecting the minimum area of the mask.

### 3.6. Photo-Realistic Rendering

In this section, we provide a detailed description of how we generate photo-realistic color-and-depth image pairs that are based on extremely realistic movements of a camera. Previous works often generate the trajectory of camera poses by the simultaneous localization and mapping (SLAM) system that is operated by a person to collect hand-held motions. After that, these poses are inserted into the scenes to synthesize new images. However, this approach’s dependence on humans to collect trajectories limits the potential scale of the dataset. Other methods synthesize images by just randomly projecting 3D objects into an arbitrary scene. However, the images that are generated by random poses are unrealistic when compared to real-world scenes. For example, the projected objects are often flying in midair or out of context [35].

**Figure 8:**
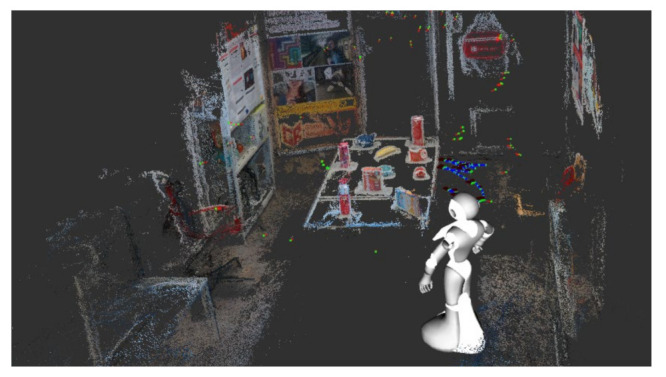
Snapshots from our simulator showing a robot synthesizing data. Green points and red lines are positions and view directions of input cameras, black lines are the view directions of the virtual camera, and the long line is the whole trajectory of the virtual camera.

Our aim is to overcome the limitations of captured datasets [9,14] and build a 3D dataset containing high-quality images generated from rich viewpoints and scales. Inspired by the low cost of producing large-scale synthetic datasets with accurate ground truth information, as well as the recent success of synthetic data used for training 6D pose estimation approaches, we use our 3D photo-realistic environment simulator [45] to generate a large number of photo-realistic color-and-depth image pairs with ground truth 6D poses for our dataset (see Figure 8).

Unlike previous methods, our trajectory generation process is automated and controllable, which is able to avoid unreasonable images and human labor. We first import the robot model that is equipped with cameras into our environment simulator. Subsequently, we move the robot to positions where the camera can capture the target object. At the same time, we record these sparse positions as our initial poses, which can be obtained by the tf package from Robot Operating System (ROS). For each pose, we randomly rotate or move the camera along its axes to obtain new poses. To make the target object visible, we set the maximum rotation angle to be less than 30 degree and the movement distance to be less than 0.2m. In this way, we are able to generate infinitely many reliable camera poses.

Our poses have three main advantages. Firstly, our poses are random, but always tracking the target object, rather than moving along a wall. Secondly, they contain a variety of movements, like that of a person collecting data. FInally, they also have limited rotational freedom that emphasizes yaw and pitch rather than roll, which is less important in 6D object pose estimation.

Based on the generated poses, the view synthesis module of our simulator that is based on image based rendering to synthesize images is used to produce photo-realistic color-and-depth image pairs. Even though we set the rotation and movement threshold to avoid synthesizing images without the target object, there are still some such images. During offline processing, we project the 3D model of the target object to synthesized images in order to check whether these images contain the target object. If not, we delete the images. In this way, we are able to make sure that all of the synthesized images satisfy our requirements.

### 3.7. Content

Based on the pipeline that is described above, we generate the RobotP dataset containing eight rich-texture, low-texture, and reflective objects recorded on two table layouts. The RobotP dataset consists of two subsets: one is a training dataset containing 3200 synthesized photo-realistic color-and-depth image pairs and the other is a testing dataset containing 1000 captured and synthesized color-and-depth image pairs. More specifically, we provide the following data:

- 6D poses for each object.
- Color and depth images with the resolution of 1280×720 in PNG.
- Segmentation binary masks and 2D bounding boxes for the objects.
- 3D point clouds with RGB color and normals for the objects.
- Calibration information for each image.

## 4. Experimental Results

### 4.1. Evaluation of Dataset Generation Approaches

We test our approaches on two scenarios (Table1, Table2) used for dataset generation, which contain a variety of objects with different sizes, shapes, textures, and occlusion.

**Figure 9:**
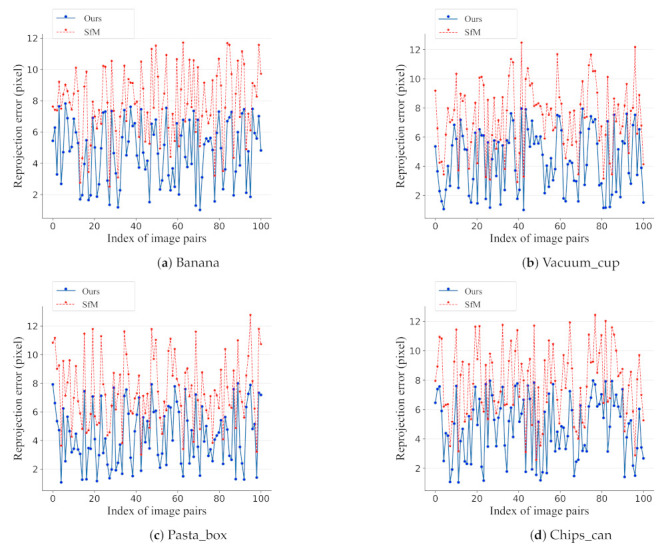
Reprojection error comparison with and without pose refinement for different objects.

**The effect of pose refinement.** We use reprojection errors to measure the accuracy of the estimated 6D pose. The reprojection error is calculated by first projecting 2D correspondences in an image to its matching image plane and then computing the pairwise distances in the image space. Figure 9 shows the reprojection errors that are calculated over 100 image pairs for different objects. We can see that, as compared with the state-of-the-art SfM [18] method, the refined poses that are produced by our approach have lower reprojection errors throughout all of the testing image pairs, which verifies the effectiveness of our pose refinement step.

**Figure 10:**
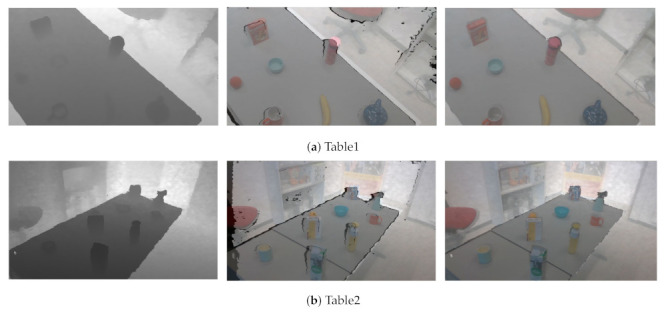
Examples of depth alignment results on table1 and table2 scenarios. The first column is the aligned depth image, the second column is the matching between captured depth and color images, and the third column is the matching between aligned depth and color images. The black color is the missing information.

**Effect of depth alignment and fusion approaches.**Figure 10 shows the depth alignment results for different color-and-depth image pairs. It can be seen that our alignment approach effectively aligns the depth image to its corresponding color image.

**Figure 11:**
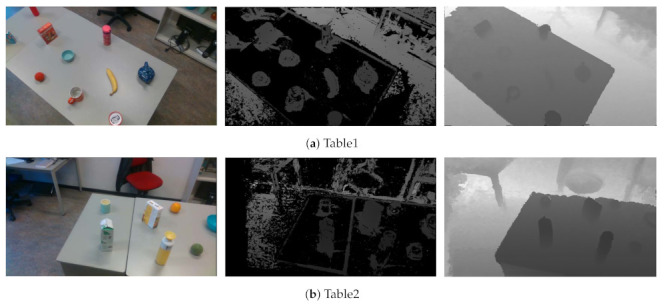
The depth fusion results on table1 and table2 scenarios. The first column are color images, and the second column are the estimated depth images by COLMAP and the third column are the depth images generated by our approach.

The main advantage of our depth fusion algorithm is its robustness towards texture-less regions. Because there is no ground truth for quantitative comparison, we provide visual comparison results, as shown in Figure 11. We can see that the performance of COLMAP degrades significantly when there is a texture-less region in the image. In contrast, our method has better performance in these regions.

**Figure 12:**
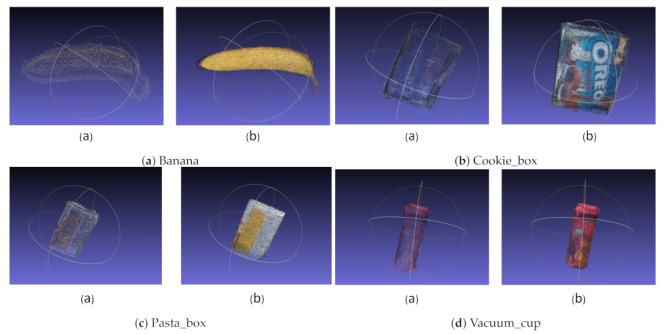
Examples of three-dimensional (3D) point clouds for the objects in our dataset. The point clouds shown in figures (a) and (b) are generated by COLMAP and our approach, respectively.

**Point clouds of objects.** We use point clouds to represent the objects in our dataset. The modeling results are shown in Figure 12. As we can see, when compared with COLMAP, our approach generates more detailed point clouds, especially for texture-less objects. For example, for the texture-less banana, our method can capture high-frequency geometric features, while the reconstruction quality of COLMAP drops significantly.

**Figure 13:**
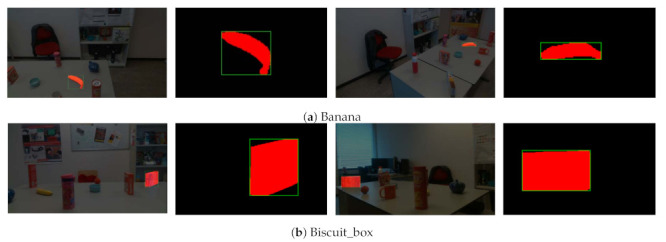
Examples of segmentation masks and bounding boxes for different objects.

**Segmentation masks and 2D bounding boxes of objects.** Based on the 3D point clouds, we generate the object mask and 2D bounding box automatically. Figure 13 shows the example results of object masks and their corresponding bounding boxes. It can be seen that the generated bounding boxes tight and stably concentrate on the objects, which demonstrates that our method produces highly accurate object masks and bounding boxes.

**Evaluation of synthesized images.** We create two subsets with 100 images from our initially captured dataset in order to quantitatively evaluate our approach. The Dense set is generated with densely captured images, while the Sparse set contains sparsely captured images. We randomly choose an image from the subset as our ground truth image and then use the other images in the subset to synthesize the chosen image. We compare our method with state-of-the-art learning-based algorithms. The peak signal-to-noise ratio (PSNR) is used to evaluate image quality, where a higher PSNR value means a better image quality. Table 3 summarizes the quantitative evaluation results.

**Figure 14:**
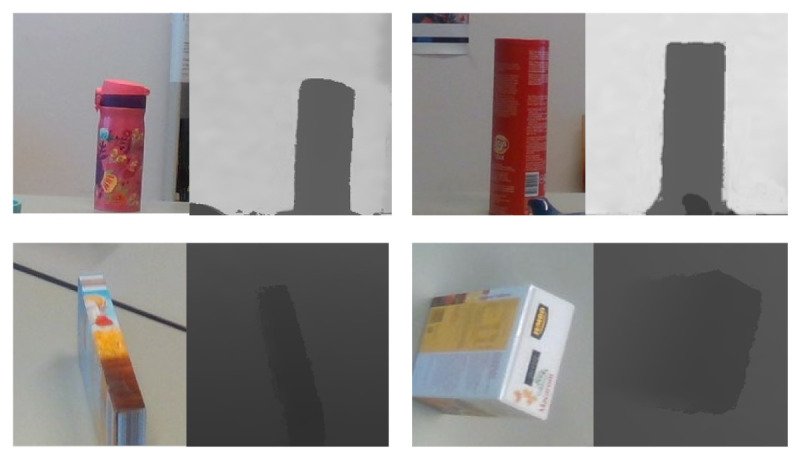
Examples of synthesized color-and-depth image pairs.

As we can see, even though the result of NeRF [48] is better when compared to other methods on Dense set, our method achieves the best performance on Sparse set. It indicates that our method is more robust to the dataset which is captured sparsely. Figure 14 shows some examples of synthesized images.

### 4.2. 6D Object Pose Estimation Challenge

Based on our dataset, we organize the Shape Retrieval Challenge benchmark on 6D object pose estimation. We test three learning-based algorithms, including DenseFusion, ASS3D, and GraphFusion, in our dataset. A more detailed description of these methods can be found in [49].

**Overall performance.** The performance of 6D object pose estimation is evaluated by ADD(-S) [9] which are the average distance metric (ADD) and the average closest point distance (ADD-S), and the area under ADD curve (AUC). A predicted pose is considered to be correct if ADD(-S) calculated with this pose is less than 10% of a model diameter, as mentioned in [9]. Table 4, Table 5 and Table 6 show the overall performance of proposed approaches.

**Figure 15:**
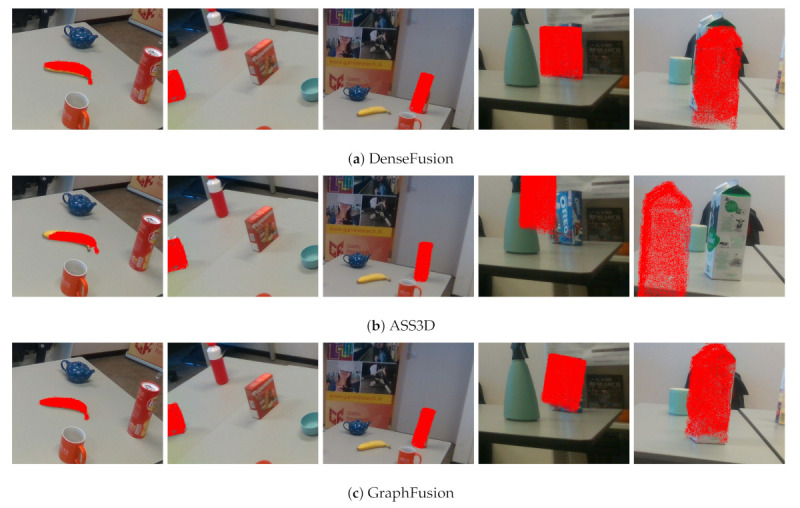
Examples of accuracy performance. Each 3D model is projected to the image plane with the estimated 6D pose.

Furthermore, in Figure 15, we also visualize the comparison results. It can be seen that DenseFusion, ASS3D, and GraphFusion provide more accurate 6D poses for colorful objects, while these approaches are less robust against dark color or reflective objects. For example, the performance of DenseFusion from the colorful biscuit box to reflective vacuum cup drops significantly ( 91% to 61% in terms of ADD). Similarly, the pose accuracy of GraphFusion from the gingerbread box with bright red color to the cookie box with a dark blue color has a large decrease, from 95% to 75%, in terms of ADD-S.

**Effect of training images.** In order to investigate the effectiveness of using photo-realistic images as training images, we use DenseFusion, ASS3D, and GraphFusion for the performance evaluation. All of the models are trained on synthesized images, and then tested on real captured and synthesized images that are never seen during training. Table 7 shows the comparison results.

It is noteworthy that knowledge learned from photo-realistic images can be successfully transferred to real-world data without domain constraints. For example, GraphFusion that is trained on synthetic data can provide accurate 6D poses for both real captured and synthesized images in terms of biscuit box (92% and 93%), respectively. This is a useful finding, as synthesizing photo-realistic images needs less hardware and does not require any human effort to capture and annotate training images.

**Time efficiency.** We compare the time efficiency among DenseFusion, ASS3D, and GraphFusion in Table 8. As can be seen, ASS3D runs the fastest among these methods. In particular, ASS3D runs four times faster than GraphFusion. This is mainly because ASS3D estimates the object pose in a single and consecutive network, which is helpful for improving the prediction speed.

## 5. Conclusions and Future Work

In this paper, we have presented the RobotP dataset, a benchmark dataset containing high-resolution color and depth images, ground truth 6D poses, segmentation masks, 2D bounding boxes, and 3D models for 6D object pose estimation. In order to build the dataset, we choose eight representative objects with the consideration of many practical issues, including cost, sizes, shapes, textures, and portability. Subsequently, we use a well-chosen 3D camera to collect data for these objects. A pose refinement approach combining local and global optimization is introduced to generate accurate ground truth 6D poses. We generate new depth images by aligning and fusing estimated depth images generated by MVS and the depth camera to further improve the quality of captured depth images. Based on the fused depth images, we produce accurate 3D models, and then we use these models to generate segmentation masks and 2D bounding boxes automatically. Besides, taking advantage of image based rendering, we synthesize a large number of photo-realistic color-and-depth image pairs with ground truth 6D poses.

Our dataset is freely distributed to research groups through the 6D object pose estimate challenge that was organized by us. It is designed to serve as a widely used benchmark dataset for robotic grasping and manipulation tasks. Our benchmark allows for evaluating and comparing pose estimation algorithms under the same standard, and it has the potential to further enrich and boost the research of 6D object pose estimation and its applications. Apart from that, our dataset can be used for other robot vision tasks, such as object detection, semantic segmentation, and depth estimation.

**Future work.** We also note some limitations of our dataset, which we hope to improve in the future. Firstly, the synthetic dataset needs to be expanded by adding more challenging objects, such as reflective and texture-less objects, and challenging conditions, such as heavy occlusion and varying lighting conditions. We plan to make object models easily integrated into a variety of robot simulation packages. When these modes are imported into a simulation environment, a variety of motion planners and optimizers can use these models as either collision or manipulation objects. Apart from that, we plan to add 4D/5D models [50,51,52] to our dataset, as 4D/5D models will benefit indoor and outdoor dynamic scene reconstruction, which plays an important role on vision-based applications, e.g., navigational systems managing moving objects.

## Figures and Tables

**Table 1:** Object datasets used for six-dimensional (6D) object pose estimation.

Dataset	Year	3D	Synthetic Data	Scenario	Novel	Available Data
Linemod [14]	2012	Yes	No	Household	Yes	RGB-D images, 3D models, object masks and bounding boxes
KIT [26]	2012	Yes	No	Household	No	2D images and 3D models
Linemod-Occluded [27]	2014	Yes	No	Household	Yes	RGB-D images, 3D models, object masks and bounding boxes
IC-MI [28]	2014	Yes	No	Household	Yes	RGB images and 3D models
IC-BIN [29]	2016	Yes	No	Household	Yes	RGB-D images and 3D models
Rutgers APC [30]	2016	Yes	No	Household	Yes	RGB-D images and 3D models
T-LESS [32]	2017	Yes	No	Industry	No	RGB-D images and 3D models
ITODD [15]	2017	Yes	No	Industry	No	Gray images and 3D models
TYO-L [37]	2018	Yes	Yes	Household	No	RGB-D images, 3D models, and object masks
YCB-Video [9]	2018	Yes	Yes	Household	No	RGB-D images, 3D models, object masks and bounding boxes
HomebrewedDB [34]	2019	Yes	Yes	Household and Industry	No	RGB-D images and 3D models
RobotP (ours)	2020	Yes	Yes	Household	Yes	RGB-D images, 3D models, object masks and bounding boxes

**Table 2:** The basic parameters of the Intel RealSense D415 camera.

Camera	Baseline	Depth FOV HD (Degree)	IR Projector FOV	Color Camera FOV	Z-Accuracy (or Absolute Error)	Module Dimensions (mm)
D415	55 mm	H: 65 ± 2/V: 40 ± 1/D: 72 ± 2	H: 67/V: 41/D: 75	H: 69 ± 1/V: 42 ± 1/D: 77 ± 1	<2%	X = 83.7/Y = 10/ Z = 4.7

**Table 3:** Quantitative evaluation of the synthesized images in terms of peak signal-to-noise ratio (PSNR) (dB).

	SM [46]	LLFF [47]	NeRF [48]	Ours
Dense	20.11	22.93	**33.25**	30.17
Sparse	11.47	14.04	21.67	**27.09**

**Table 4:** Quantitative evaluation of the 6D pose in terms of average distance metric (ADD).

	Banana	Biscuit_Box	Chips_Can	Cookie_Box	Gingerbread_Box	Milk_Box	Pasta_Box	Vacuum_Cup	MEAN
DenseFusion	**0.86**	0.91	0.56	**0.62**	0.87	0.50	0.77	0.61	0.71
ASS3D	0.70	0.78	0.75	0.49	0.63	0.58	0.63	**0.65**	0.65
GraphFusion	0.83	**0.93**	0.69	0.61	**0.90**	**0.66**	**0.84**	0.63	**0.76**

**Table 5:** Quantitative evaluation of the 6D pose in terms of average closest point distance (ADD-S).

	Banana	Biscuit_Box	Chips_Can	Cookie_Box	Gingerbread_Box	Milk_Box	Pasta_Box	Vacuum_Cup	MEAN
DenseFusion	0.86	0.95	0.94	0.74	0.94	**0.81**	0.91	0.90	0.88
ASS3D	0.75	0.88	0.85	0.66	0.86	0.62	0.72	0.75	0.76
GraphFusion	**0.87**	**0.96**	**0.97**	**0.75**	**0.95**	0.77	**0.96**	**0.97**	**0.90**

**Table 6:** The 6D pose estimation accuracy in terms of the area under ADD curve (AUC).

	Banana	Biscuit_Box	Chips_Can	Cookie_Box	Gingerbread_Box	Milk_Box	Pasta_Box	Vacuum_Cup	MEAN
DenseFusion	**0.77**	0.77	0.74	**0.67**	0.76	0.66	0.74	0.71	0.72
ASS3D	0.66	0.74	0.72	0.56	0.71	0.51	0.61	0.64	0.64
GraphFusion	0.75	**0.79**	**0.76**	0.66	**0.78**	**0.67**	**0.77**	**0.74**	**0.74**

**Table 7:** The 6D pose estimation accuracy in terms of ADD using different input images.

	Real			Synthetic		
	DenseFusin	ASS3D	GraphFusion	DenseFusin	ASS3D	GraphFusion
banana	0.85	0.70	0.84	0.86	0.70	0.82
biscuit_box	0.91	0.77	0.92	0.91	0.78	0.93
chips_can	0.55	0.75	0.69	0.57	0.74	0.69
cookie_box	0.61	0.48	0.60	0.63	0.49	0.61
gingerbread_box	0.88	0.63	0.91	0.87	0.63	0.90
milk_box	0.51	0.57	0.68	0.49	0.58	0.66
pasta_box	0.78	0.61	0.83	0.76	0.64	0.84
vacuum_cup	0.59	0.65	0.64	0.62	0.64	0.63

**Table 8:** Comparison of the computational run time among different approaches (second per frame).

	Banana	Biscuit_Box	Chips_Can	Cookie_Box	Gingerbread_Box	Milk_Box	Pasta_Box	Vacuum_Cup	MEAN
DenseFusion	0.03	0.03	0.03	0.03	0.03	0.03	0.03	0.03	0.03
ASS3D	**0.01**	**0.01**	**0.01**	**0.01**	**0.01**	**0.01**	**0.01**	**0.01**	**0.01**
GraphFusion	0.04	0.04	0.04	0.04	0.04	0.04	0.04	0.04	0.04

## Data Availability

The dataset is publicly available at: https://yhldrf.github.io/Datasets.github.io/ (accessed on 19 December 2020). If interested in using it, we encourage users to contact the correspondence author for data accessibility.
